# Description of *Nothotylenchus savadkoohensis* n. sp. (Rhabditida, Anguinidae) from Iran based on morphological and molecular data

**DOI:** 10.2478/jofnem-2024-0015

**Published:** 2024-04-22

**Authors:** Soheila Rezaei, Ebrahim Pourjam, Mohammad Reza Atighi, Majid Pedram

**Affiliations:** Department of Plant Pathology, Faculty of Agriculture, Tarbiat Modares University, Tehran, Iran

**Keywords:** D2–D3 LSU rDNA, ITS, morphometrics, new species, phylogeny, taxonomy

## Abstract

*Nothotylenchus savadkoohensis* n. sp. was recovered from rotten wood samples of an unidentified forest tree in the Mazandaran province and described herein. It is mainly characterized by an elongated conoid tail ending in a sharply pointed tip and four lines in the lateral field. Females of the new species have 379–662 μm long bodies with 5.8–6.9 μm long stylets ending in fine posteriorly sloping knobs, the metacorpus not valvate, the pharyngeal bulb slightly overlapping the intestine, and the vulva at 76.5–84.0% of body length. Males are also common and have 13.0–14.5 μm long spicules and bursa cloacal. By having an elongated conoid tail and four lines in the lateral field, the new species comes close to four known species, namely *N. acris, N. acutus, N. antricolus*, and *N. truncatus*. The morphological differences between the new species and the abovementioned species are discussed. The new species was sequenced for its D2–D3 segment of LSU and ITS rDNA regions. In the LSU phylogenetic tree, the currently available LSU sequences of the genus *Nothotylenchus* occupied distant placements from each other and the LSU sequence of the new species formed clade with a sequence assigned to *Neotylenchus* sp. In ITS phylogeny, the newly generated sequence of the new species formed a clade with a clade that includes sequences of *Ditylenchus* sp. and *Neomisticius platypi* and *N. variabilis*.

The genus *Nothotylenchus* Thorne, 1941, a member of the family Anguinidae Nicoll, 1935, is mainly delimited by non-valvate and non-muscular fusiform to slightly swollen metacorpus (Siddiqi, 2000; Andrássy, 2007). It is further characterized by fungal feeding behavior and pharyngeal glands not forming a long overlap over the intestine with glands nuclei located anterior to pharyngo-intestinal junction (Subbotin and Riley, 2012). In the last extensive taxonomic study, 41 species have been regarded as valid under the genus (Hashemi and Karegar, 2020). Most *Nothotylenchus* species, especially those in old literatures, have been established based upon classic criteria and molecular data of type or topotype populations of them are currently lacking. On the other hand, descriptions of several recently established species and recent taxonomic studies of previously known species include molecular data (e.g., Jalalinasab et al., 2018; Esmaeili et al., 2016, 2017; Munawar et al., 2022). Based on available data, the sequenced species of the genus for their ribosomal DNA, occupy distant placements in corresponding phylogenetic trees, indicating the genus is not monophyletic (Jalalinasab et al., 2018; Munawar et al., 2022).

The species *Nothotylenchus andrassyi* (amendment to *N. andrassy*) Jalalinasab, Hosseini & Heydari 2018, *N. brzeskii* Hashemi & Karegar, 2020, *N. geraerti* Kheiri, 1971, *N. persicus* Esmaeli, Heydari, Castillo & Palomares-Rius, 2016, *N. phoenixae* Esmaeili, Heydari & Ye, 2017, *N. siddiqii* Hashemi & Karegar, 2020 and *N. tuberosus* Kheiri, 1971 have originally been described from Iran. Furthermore, the species *N. acris* Thorne, 1941, *N. acutus* Khan, 1965, *N. adasi* Sykes, 1980, *N. affinis* Thorne, 1941, *N. basiri* Khan, 1965, *N. hexaglyphus* Khan & Siddiqi, 1968, *N. medians* Thorne & Malek, 1968 occur in Iran as well (Hashemi and Karegar, 2020).

Since 2020, no extra species have been added to the genus. During recent samplings and taxonomic studies in Mazandaran province, a species of the genus was recovered from the rotten wood of an unidentified forest tree. After comparing it with all valid species of the genus listed by Hashemi and Karegar (2020), the detailed morphological studies revealed that it belongs to a new species. Thus, the present study aims to describe this species using morphological and molecular data.

## Materials and methods

### Soil and wood sampling, nematode extraction, mounting, and permanent slide preparing

A number of 45 soil, wood, and bark samples were collected from the natural forests of the city of Savadkooh, Mazandaran province. Nematodes were extracted from these samples using the tray method (Whitehead and Hemming, 1965). A population of *Nothotylenchus* sp. was recovered from rotten wood samples of an unidentified forest tree. The collected specimens were killed by adding boiling 4% formalin solution, transferred to anhydrous glycerin, mounted on permanent microscopic slides according to De Grisse (1969), and studied with a Nikon Eclipse E600 optical microscope. Photos were taken using an Olympus DP72 digital camera connected to an Olympus BX51 light microscope powered with differential interference contrast (DIC) view. Drawings were made using a drawing tube attached to the microscope and digitally drawn using CorelDRAW® software version 2020.

### DNA extraction, PCR, and sequencing

Three females were selected for this purpose. The specimens were studied in temporary slides and photographed. Each specimen was transferred into a small drop of TE buffer (10 mM Tris-Cl, 0.5 mM EDTA; pH 9.0, Qiagen) on another clean slide and squashed using a cover slip with the aid of the pressure of a pipette tip. The suspension was collected by adding 15 μL of the TE buffer. Three DNA samples were made in this manner and stored at −20°C until used as PCR templates. Primers for LSU rDNA D2–D3 amplification were forward primer D2A (5′-ACAAGTACCGTGAGGGAAAGT-3′) and reverse primer D3B (5′-TCGGAAGGAACCAGCTACTA-3′) (Nunn, 1992). The primers rDNA1 (5′-TTGATTACGTCCCTGCCCTTT-3′) (Subbotin et al., 2000) and TW81 (5′-GTTTCCGTAGGTGAACCTGC-3′) (Joyce et al., 1994) were used to amplify ITS rDNA. Forward 1096F (5′-GGTAATTCTGGAGCTAATAC-3′) and reverse 1912R (5′-TTTACGGTCAGAACTAGGG-3′) and forward 1813F (5′-CTGCGTGAGAGGTGAAAT-3′) and reverse 2646R (5′-GCTACCTTGTTACGACTTTT-3′) primers were used to amplify the SSU rDNA (Holterman et al., 2006). PCRs were performed according to Jahanshahi Afshar et al. (2019) and the successfully amplified products were directly sequenced in both directions using the same primers used in PCR with an ABI 3730XL sequencer (Pishgam corporation, Tehran, Iran). The newly generated sequences were deposited into the GenBank database under the accession numbers PP174320 for D2–D3 expansion segments of LSU and PP174321 for ITS rDNA.

#### Phylogenetic analyses

The newly obtained LSU rDNA D2–D3 sequence of the new species (two identically aligned same-sized sequences were generated, and only one of them was used) was compared with those of other nematodes already deposited into the database. A number of 33 D2–D3 sequences of representatives of superfamily Sphaerularioidea Lubbock, 1861 including anguinid and sphaerulariid taxa (Decraemer and Hunt, 2013) and two sequences belonging to classic rhabditid taxa (for accession numbers, genera and species names, see the tree) were selected to reconstruct the LSU phylogenetic tree. The aligning of the LSU dataset was performed using the Q-INS-i algorithm of online version of MAFFT version 7 (http://mafft.cbrc.jp/alignment/server/) (Katoh & Standley, 2013). The Gblocks program (http://phylogeny.lirmm.fr/phylo_cgi/one_task.cgi?task_type=gblocks) with all three less stringent parameters was used for post-editing of this alignment, *i.e.*, to eliminate poorly aligned regions or divergent positions. The most appropriate model of nucleotide substitution for this dataset was selected using Akaike information criterion (AIC) and MrModeltest 2 (Nylander, 2004). The general reversible model, including a gamma distribution of rates across sites, and a proportion of invariant sites (GTR + G + I) was selected and used for analysis of this dataset. The newly generated ITS sequence of the new species was compared with currently available relevant sequences in GenBank database. The sequences with a high percentage of coverage were selected (six ingroup and one outgroup sequence; for genera and species names and accession numbers, see the tree). The alignment of the ITS dataset was performed using ClustalX2 (http://www.clustal.org/), and the resultant alignment was manually edited using MEGA6 (Tamura et al., 2013). The Hasegawa-Kishino-Yano model, including a gamma distribution of rates across sites (HKY + G), was selected for the ITS dataset using the abovementioned method.

Bayesian inference (BI) was performed using MrBayes v3.1.2 (Ronquist & Huelsenbeck, 2003), running the chains for 3 × 10^6^ generations for LSU and 1 × 10^6^ generations for the ITS dataset. After removing 25% of the retrieved samples by burning them, the remaining samples were stored for additional analyses. Markov chain Monte Carlo (MCMC) method within a Bayesian framework was used to estimate Bayesian posterior probabilities (BPPs) of phylogenetic trees (Larget & Simon, 1999) using the 50% majority rule. Tracer v1.5 software (Rambaut & Drummond, 2009) was used to visualize the results of each run and to verify the effective sample size for each parameter.

## Results

*Nothotylenchus savadkoohensis* n. sp.

Figs. 1&2.

**Figure 1:**
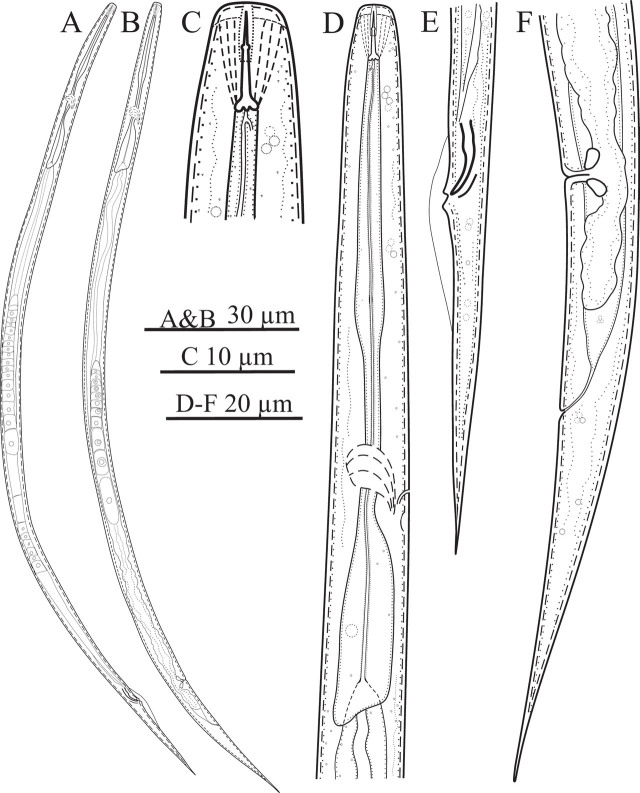
Line drawings of *Nothotylenchus savadkoohensis* n. sp. A, B: Male and female entire body; C: Anterior body region; D: Female pharyngeal region; E: Male posterior body region; F: Female posterior body region.

**Figure 2:**
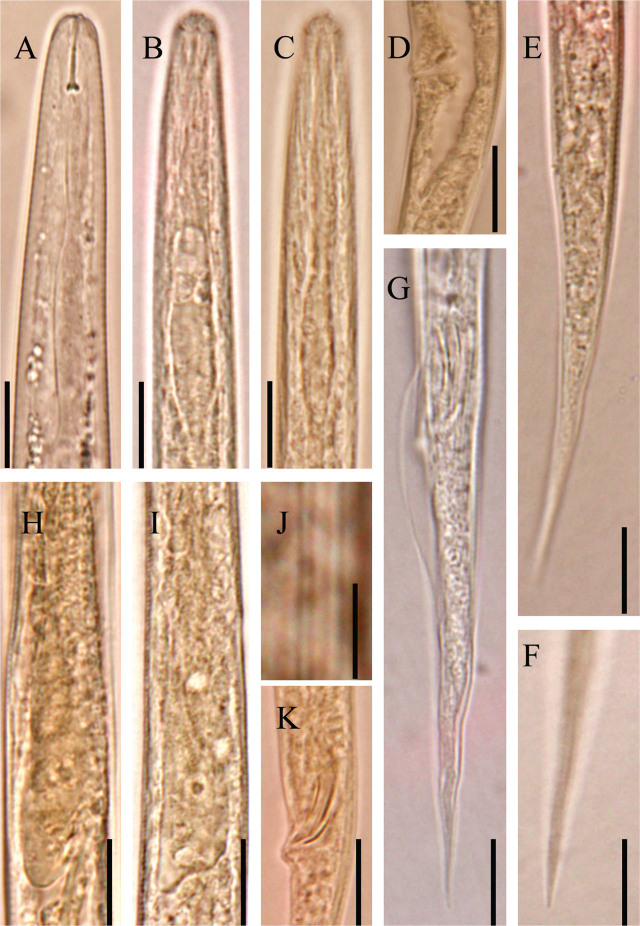
Light micrographs of *Nothotylenchus savadkoohensis* n. sp. A: Anterior body region; B & C: Non-muscular metacorpus; D: Vulva region and postvulval uterine sac; E: Female posterior body region (tail); F: Female tail tip; G: Male posterior body region and bursa; H & I: Short overlapping of pharyngeal bulb; J: Lateral field; K: Spicules (Scale bars: A-I, K=10 μm, J=5 μm).

Measurements, see Table 1

**Table 1:** Morphometrics of *Nothotylenchus savadkoohensis* n. sp. All measurements are in μm and in the form: mean ± standard deviation (range).

**Character**	**Females**		**Males**

	**Holotype**	**Paratypes**	**Paratypes**
n	-	12	4
L	485	526±80 (379–662)	496.3±8.5 (485–505)
a	37.3	38.4±5.2 (27.1–47.3)	37.9±2.4 (34.6–40.4)
b	4.8	5.4±0.7 (4.2–6.3)	5.2±0.2 (5.0–5.4)
c	9.7	11.1±1.4 (8.4–13.9)	10.1±0.9 (9.2–11.2)
c′	6.7	6.1±0.6 (4.9–7.3)	6.3±0.9 (5.0–6.9)
V	79.4	81.8±2.3 (76.5–84.0)	-
Anterior end to vulva	385	426±74 (290–550)	-
Lip region height	3	2.5±0.5 (2.0–3.2)	2.3±0.2 (2.0–2.5)
Lip region width	5	5.0±0.3 (4.8–5.7)	5.0±0.1 (4.8–5.1)
Stylet conus	2	2.1±0.2 (2.0–2.5)	2.2±0.1 (2.0–2.3)
Stylet	6	6.2±0.4 (5.8–6.9)	6.21±0.4 (5.6–6.5)
m	33.3	33.8±2.2 (30.8–37.1)	34.9±1.9 (32.3–36.5)
Anal/cloacal diameter	7.5	8.5±0.5 (8–9)	8.0±0.8 (7.2–9.0)
Maximum body with	13	13.6±0.9 (12–15)	13.1±0.6 (12.5–14.0)
Vulval body width	11	11.9±1.2 (10–14)	-
Anal body width	7.5	7.9±3.4 (7.5–9.0	7.7±0.6 (7–8)
Excretory pore	77	76.3±3.4 (68–80)	72.7±1.8 (71.0–74.5)
Hemizonid	71	70.0±1.5 (67–72)	70.7±2.1 (68.0–72.6)
Pharynx	102	97.6±5.6 (90–105)	96.5±5.2 (90–102)
Nerve ring	55	56.6±2.5 (53–62)	60±5 (53–65)
Overlap length	4.9	4.8±0.2 (4.5–5.2)	3.0±0.7 (4.0–4.8)
Postvulval uterine sac (PUS)	15	18.7±3.1 (15–26)	-
Vulva to anus	45	45.4±5.7 (40.0–55.5)	-
Tail	50	47.8±4.4 (42–55)	49.5±3.3 (45–53)
Spicules	-	-	13.5±0.4 (13.0–14.5)
Gubernaculum	-	-	4.8±0.2 (4.5–5.0)

### Description

#### Female

Body slender, ventrally slightly arcuate after heat relaxation, slightly narrowing toward both extremities, more so towards posterior end by having an elongated conoid tail with narrow distal end. Cuticular annulation fine. Lateral field with four incisures. Lip region low, anteriorly almost flat, the cephalic framework weak. Stylet fine, its conus about 34% of the total stylet length, with fine knobs, sloping backward. Procorpus slender, widened at about middle of the pharynx, metacorpus not valvate, isthmus narrow, joining to pharyngeal bulb, slightly overlapping intestine dorsally. Nerve ring enveloping isthmus slightly anterior to pharyngeal basal bulb. Hemizonid close to tip of nerve ring. Intestine simple, rectum and anus functional. Reproductive system monodelphic-prodelphic, composed of outstretched ovary with oocytes arranged in single row, oviduct tubular, spermatheca elongate and including spheroid sperm, crustaformeria with no clearly seen cells in each row, uterus, vagina perpendicular to body axis, vulva a small transverse slit, and postvulval uterine sac (PUS) about 1.3 times corresponding body width long. The sphincter encircling vagina triangle-shape at cross section. The distance from vulva to anus is about four times vulval body width. Tail is elongated and conical, almost straight, with a sharply pointed tip.

#### Male

General morphology similar to that of the females, except slightly shorter body and characters related with sexuality. Reproductive system monorchic, testis single, outstretched. Spicules tylenchoid, slightly sclerotized, ventrally arcuate, gubernaculum simple. Bursa cloacal. Tail elongate conoid, uniformly narrowing toward distal end, with sharp tip.

#### Etymology

The specific epithet refers to the city of Savadkooh from where the new species was recovered.

#### Type host and locality

New species was recovered from rotten wood samples of an unidentified forest tree, collected from forests of Mazandaran province, city of Savadkooh in northern Iran (GPS coordinates; 36°12.795N, 052°55.581E).

#### Type materials

Holotype female, 12 paratype females, and four paratype males in 16 slides were deposited at WaNeCo collection, Wageningen, The Netherlands (http://www.waneco.eu/). The LSID code for this publication is: urn:lsid:zoobank.org:pub:C9E20B3E-D502-4A31-957D-F5B81C3B68FD.

#### Diagnosis and relationships

*Nothotylenchus savadkoohensis* n. sp. is mainly characterized by having four lines in lateral fields and an elongated conoid tail with sharply pointed tip. It is further characterized by 379–662 μm long females, having 5.8–6.9 μm long stylet with fine posteriorly sloping knobs, no valvate metacorpus, pharyngeal bulb slightly overlapping intestine dorsally, vulva at 76.5–84.0% of body length and males with cloacal bursa. By having an elongated conoid tail and four lines in the lateral fields, the new species comes close to four known species of the genus namely *Nothotylenchus acris, N. acutus* Khan, 1965, *N. antricolus* Andrássy, 1961 and *N. truncatus* Eliashvili & Vacheishvili, 1980. The new species could be separated from them as follow:

From *N. acris* by a shorter body (379–662 vs 900 μm) and tail shape (elongated conoid narrowing toward the distal region, sharply pointed at tip vs elongate conoid, thicker at distal region).

From *N. acutus* by greater V (76.5–84.0 vs 70–76.3), shorter stylet (5.8–6.9 vs 7–9 μm), greater a (27.1–47.3 vs 22–28) and tail shape (elongate conoid narrowing toward distal region, sharply pointed at tip vs elongate conoid, thicker at distal region).

From *N. antricolus* by greater c (8.4–13.9 vs 6.7–6.9), greater V (76.5–84.0 vs 70.7–72.4), smaller c′ (4.9–7.3 vs 9.6–10.0), longer PUS (longer than corresponding body with vs shorter), and difference in tip shape (elongate conoid, narrowing toward distal region, sharply pointed at tip vs elongate conoid, thicker at distal region).

From *N. truncatus* by greater V (76.5–84.0 vs 57–66), greater c (8.4–13.9 vs 6.3–7.4), shorter stylet (5.8–6.9 vs 11.0–12.5 μm), and difference in tail tip shape (sharply pointed vs rounded).

#### Molecular analyses and phylogeny

**Figure 3:**
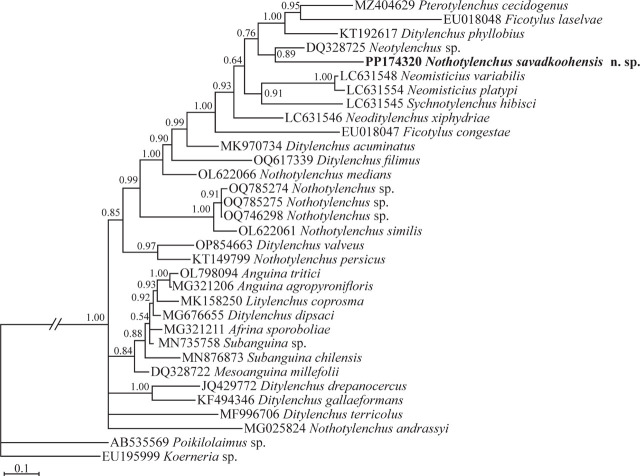
Bayesian 50% majority rule consensus tree inferred from D2–D3 expansion region of LSU rDNA sequence of *Nothotylenchus savadkoohensis* n. sp. from Mazandaran province under the GTR + G + I model. Bayesian posterior probability (BPP) values >0.50 are given for appropriate clades. The newly generated sequence of the new species is in bold font.

Several efforts to get sequences of SSU rDNA for the new species using formerly cited primer pairs failed. The PCRs for amplification of D2–D3 region of LSU rDNA of two female specimens were successful. The two LSU sequences obtained from two female specimens were identical, and as the result, one sequence was deposited into the GenBank database under the accession number PP174320. This sequence is 558 nt long. The BLAST search using this sequence revealed its identity with all currently available sequences as less than 91%. Fig. 3 represents the Bayesian phylogenetic tree reconstructed using the LSU dataset. In this tree, currently available D2–D3 sequences of the genus *Nothotylenchus* have occupied distant placements, and the D2–D3 sequence of the new species has formed a clade with a sequence assigned to *Neotylenchus* sp. (DQ328725). The morphological data of this isolate are, however, not available. Based on the currently resolved topology, the genus *Nothotylenchus* is polyphyletic according to currently available data.

**Figure 4:**
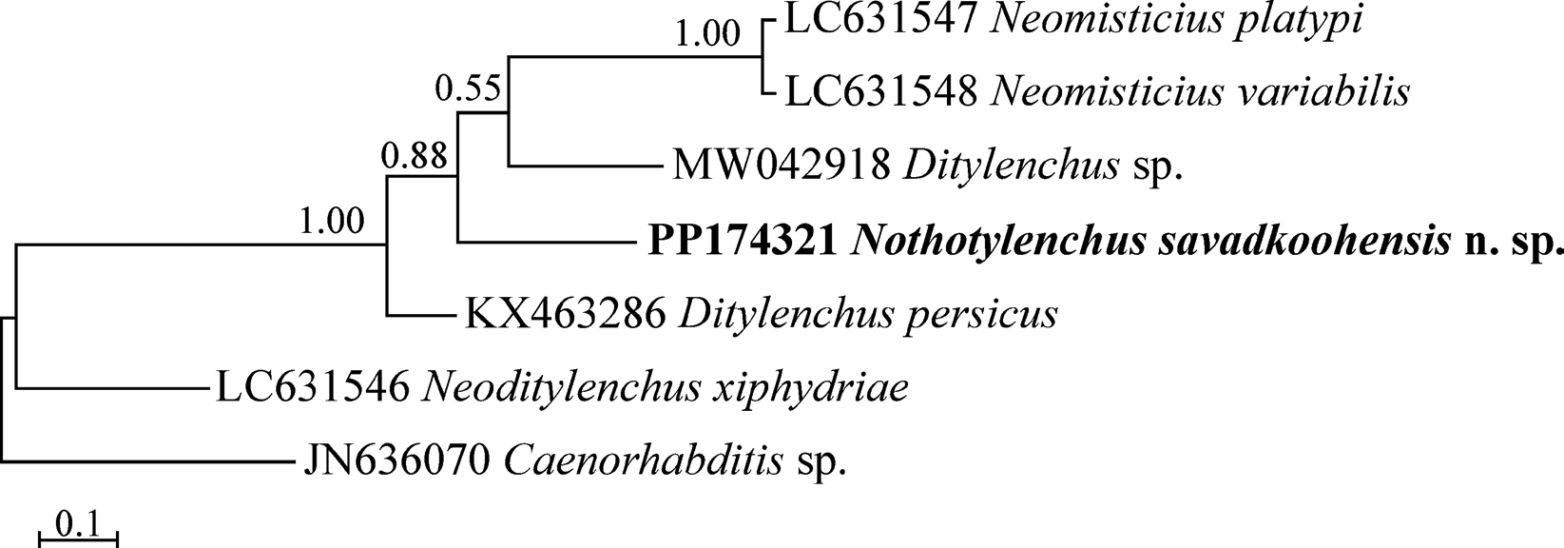
Bayesian 50% majority rule consensus tree inferred from ITS rDNA sequence of *Nothotylenchus savadkoohensis* n. sp. from Mazandaran province under the HKY + G model. Bayesian posterior probability (BPP) values >0.50 are given for appropriate clades. The newly generated sequence of the new species is in bold font.

The partial ITS sequence of the new species (PP174321) was 394 nt long. The BLAST search using this sequence revealed the identity of this sequence with sequences having high (above 75%) coverage ranges from 91–94%. Fig. 4 represents the phylogenetic tree reconstructed using the ITS dataset. In this tree, the newly generated sequence of the new species has formed a clade with a clade that includes sequences of a *Ditylenchus* sp. (MW042918) and two sequences of *Neomisticius platypi* Masuya & Hamaguchi, 2021 and *N. variabilis* Kanzaki, Masuya & Hamaguchi, 2021 (LC631547 and LC631548).

## Discussion

Validity of the genus *Nothotylenchus* is now well established (Siddiqi, 2000; Andrássy, 2007, Subbotin and Riley, 2012; Hashemi and Karegar, 2020). In general morphology, it looks similar to *Ditylenchus* Filipjev, 1936, but could be separated from it using a combination of characters *viz*. a non-valvate and non-muscular metacorpus and pharyngeal glands nuclei located anterior to pharyngo-intestinal junction (Siddiqi, 2000; Andrássy, 2007, Subbotin and Riley, 2012). From the known species of *Nothotylenchus*, six species were originally described for Iran, and seven already known species, however, occur in the country. The recovery of the newly described species during the present study indicates that the diversity of the genus in Iran could still be higher. The phylogenetic relationships of the presently described new species with other relevant species and genera were reconstructed using two LSU D2–D3 and ITS rDNA markers. The general topology of the currently reconstructed LSU tree is in agreement with the previous LSU phylogenies (e.g., Munawar et al., 2022), showing the available sequences of *Nothotylenchus* occupy distant placements in the tree, and the genus is not monophyletic. Thus, an integrative approach using both morphological and molecular data should be exploited while assigning species into this genus. The relevant sequences used in ITS phylogeny also yielded in cladogenesis events, showing this marker could be used in future molecular taxonomic studies on the genus, as commonly used for other anguinid taxa (e.g. Vovlas et al., 2016; Barrantes-Infante et al., 2018).
